# Iron oxide nanoflowers encapsulated in thermosensitive fluorescent liposomes for hyperthermia treatment of lung adenocarcinoma

**DOI:** 10.1038/s41598-022-12687-3

**Published:** 2022-05-24

**Authors:** Maria Theodosiou, Elias Sakellis, Nikos Boukos, Vladan Kusigerski, Beata Kalska-Szostko, Eleni Efthimiadou

**Affiliations:** 1 Laboratory of Inorganic Chemistry, Department of Chemistry, National and Kapodistrian University of Athens, Athens, Greece; 2 Institute of Nanoscience and Nanotechnology, National Center for Scientific Research “Demokritos”, Athens, Greece; 3 Institute of Nuclear Sciences Vinca, University of Belgrade, Belgrade, Republic of Serbia; 4 Faculty of Chemistry, University of Białystok, Białystok, Poland

**Keywords:** Cancer, Drug discovery, Chemistry, Materials science

## Abstract

Magnetic hyperthermia (MHT) is in the spotlight of nanomedical research for the treatment of cancer employing magnetic iron oxide nanoparticles and their intrinsic capability for heat dissipation under an alternating magnetic field (AMF). Herein we focus on the synthesis of iron oxide nanoflowers (Nfs) of different sizes (15 and 35 nm) and coatings (bare, citrate, and Rhodamine B) while comparing their physicochemical and magnetothermal properties. We encapsulated colloidally stable citrate coated Nfs, of both sizes, in thermosensitive liposomes via extrusion, and RhB was loaded in the lipid bilayer. All formulations proved hemocompatible and cytocompatible. We found that 35 nm Nfs, at lower concentrations than 15 nm Nfs, served better as nanoheaters for magnetic hyperthermia applications. In vitro*,* magnetic hyperthermia results showed promising therapeutic and imaging potential for RhB loaded magnetoliposomes containing 35 nm Nfs against LLC and CULA cell lines of lung adenocarcinoma.

## Introduction

Lung cancer is the number one cause of cancer-related deaths worldwide, of which over 80% is identified as non-small cell lung cancer^1,2^. Conventional treatments for cancer, like chemotherapy, radiation, or surgery, have numerous side effects for the patient (nausea, hair loss, intestinal dysfunction, skin irritability), entailing risks of drug resistance, collateral damage of healthy tissue, inefficiency against metastasis, and tumor reoccurrence^3^.

Nanotechnological advances of the last 30 years have paved the way to a prominent alternative treatment; magnetic fluid hyperthermia therapy (MHT). The basis of MHT is classic hyperthermia (HT) which refers to local or whole-body temperature increase between 40–45 °C where tumor tissues are susceptible while at this range, neighboring healthy tissue can sustain heat^4^. Although the concept of HT dates back to Hippocrates, modern medicine’s clinical applications start in the late nineteenth century employing external heating sources and continue to date as adjunctive treatments to chemotherapy and radiation^5,6^. The pathophysiologic characteristics of tumor areas, including a leaky vasculature and disorganized cellular architecture, can hinder localized heat dissipation in cancer tissue^7^. Thus, the need for precise application of HT to tumor areas led to the first experimental clinical approach of MHT by Gilchrist et al.; in 1957, he exploited the inherent ability of magnetic nanoparticles to dissipate heat under the application of an alternating magnetic field (AMF)^8–12^. Since then, superparamagnetic iron oxide nanoparticles (SPIONs) of different sizes, shapes (spheres, cubes, rods, flowers, disks) and crystalline structures (magnetite, maghemite, hematite) have been studied for their potential applications in nanomedicine as MHT heating mediators^13–16^. Upon applying an AMF over a ferrofluid consisting of SPIONs, the magnetic energy overcomes the anisotropy energy causing thermal energy dissipation, which is quantified in terms of specific absorption rate (SAR)^17,18^. The heat generation mechanism by SPIONs depends on susceptibility losses attributed to the Brownian motion of the particles within the dispersant and the Néel relaxation processes, which are inextricably related to size and shape anisotropy factors^19,20^. Accumulation of biocompatible magnetic nanoparticles to tumor areas by active or passive targeting and subsequent MHT application leads to cellular distortion in the tumor microenvironment and ultimately to cancer cell death, through either apoptosis or necrosis depending on the type of cells, the localization of the nanoparticles and the temperature^21–23^.

The biocompatibility and bioavailability of the administered ferrofluids are of utmost importance to reserve minimal systemic toxicity and ensure prolonged circulation before clearance effects. Coating with polymers or small molecules, like citrate, are common paths used in nanochemistry to ameliorate the biocompatibility profile of SPIONs^24^. Another alluring approach is to encapsulate them in or between lipid bilayers and formulate magnetoliposomes^25^. Lipid vesicles have an innate affinity towards biological membranes rendering them and their load biocompatible and excellent delivery agents^26^. Emphasis should be given to selecting proper lipids for magnetoliposomes destined for MHT. Thermosensitive liposomes consist of lipids with the potential of gel-to-liquid phase transition above a critical temperature (T_c_) characteristic of each lipid type^27^. When thermally triggered liposomes enter the liquid phase, they maintain their structure while at the same time the permeability of the lipid bilayer is increased, a property that allows the release of their payload (drugs, small molecules, mRNA)^7^. Liposomal engulfment of magnetic nanoparticles increases their stealth and blood circulation time while they can be used as drug delivery agents combining MHT with chemotherapy in a controllable way^28^.

To this end, we studied iron oxide nanoflowers (Nfs) as novel heating mediators for MHT with intriguing geometry. In literature, Nfs synthesized via the polyol method demonstrate excellent colloidal stability over time and exhibit superior SAR values owing to their cluster-like shape^29–31^. This study aims to compare the properties of different sizes and coatings of Nfs in terms of stability, magnetothermal responsiveness, and in vitro behavior (cytotoxicity and cellular localization) with or without MHT. For this purpose, we synthesized and compared 15 and 35 nm Nfs -bare, citrate coated, or encapsulated in thermosensitive liposomes- for their colloidal stability and their potential uptake by two different lung cancer cell lines (LLC and CULA). To create a fast and rigorous protocol for simultaneous cellular imaging and therapy, we attempted coating or loading the nanoformulations with Rhodamine B (RhB), an inexpensive red fluorescent laser dye with high quantum yield and photostability. Interestingly, various rhodamine dyes and their derivatives have been investigated for their potential as anticancer agents^32,33^. Thus, their incorporation within nanocarriers can increase their theranostic potential. Hence, we contrasted the in vitro behavior of Nfs and magnetoliposomes to the corresponding nanoformulations containing RhB to assess their therapeutic and imaging potential under MHT.

## Results and discussion

### Synthesis of iron oxide nanoflowers

**Figure 1:**
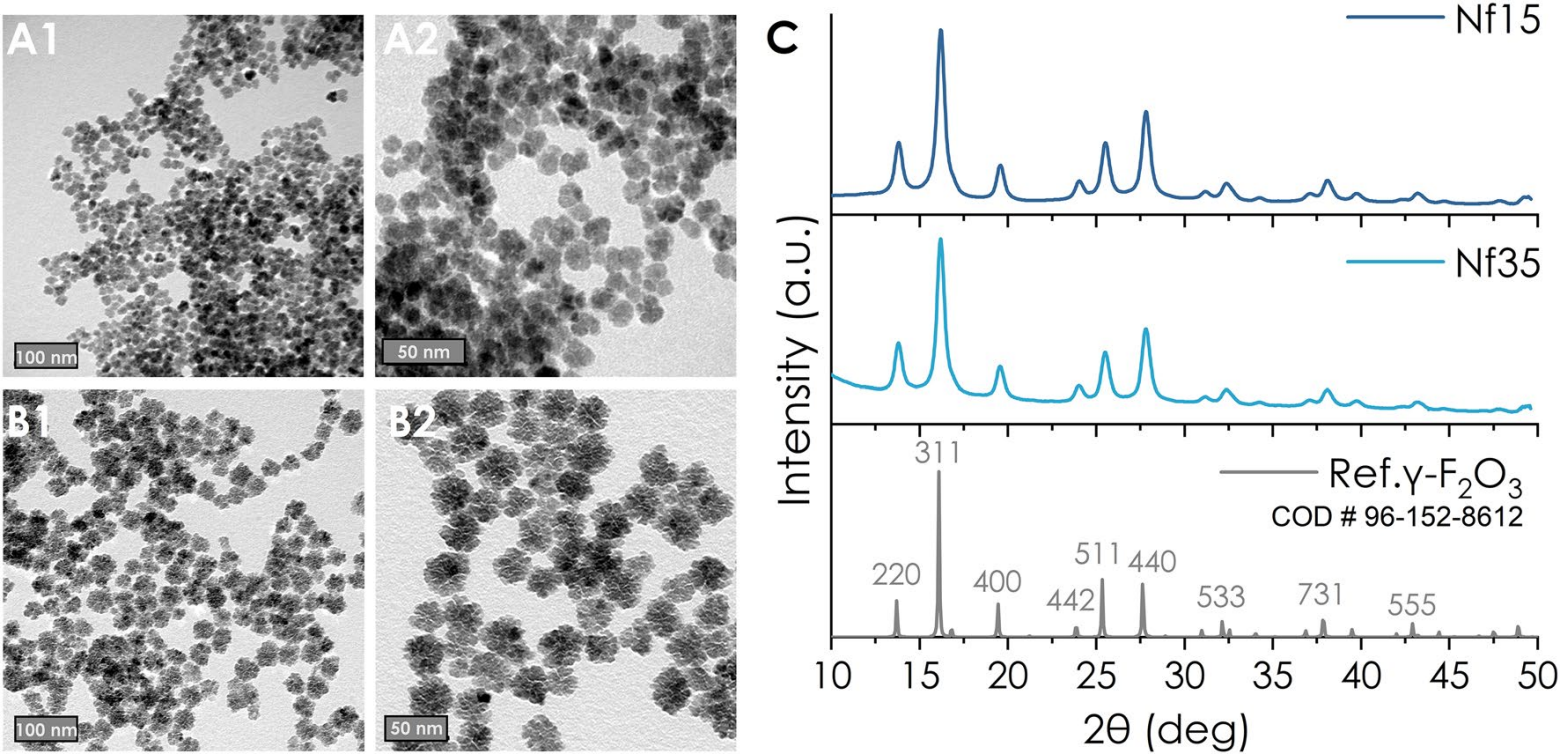
TEM images of (**A1-2**) Nf15 and (**B1-2**) Nf35 (scale bars at 50 and 100 nm), (**C**) X-ray diffraction spectrum of Nf15 and Nf35 compared to maghemite standard.

Iron oxide nanoflowers (Nfs) were synthesized by reduction of hydrate iron salts in a 1:1 v/v mixture of N-methyldiethanolamine (NMDEA) and diethylene glycol (DEG) at 210 °C at a heating rate of 2 °C/min^31,34^. NMDEA and DEG act as reducing agents and surfactants mediating the formation of flowerlike multicore iron oxide nanoparticles with narrow size distribution and exceptional colloidal stability^35^. We produced two different sizes of Nfs by varying the reaction time at 210 °C; 30 min for 15 nm (Nf15) and 60 min for 35 nm (Nf35). TEM images (Fig. 1A,B) revealed consistent morphology and lognormal size distribution (Nf15 d_TEM_: 15.1 ± 2.8 nm and Nf35 d_TEM_: 35.0 ± 3.8 nm) (Supplementary Fig. S1A).

The oxidation into maghemite (γ-Fe_2_O_3_) crystalline structure is confirmed by XRD (Fig. 1C), in accordance with literature^36^. Maghemite is the preferable form of iron oxide for biological applications because it has less reactive sites than magnetite, thus minimizing the production of harmful reactive oxygen species in the healthy cellular microenvironment^37–40^. The crystallite size-determined by Scherrer’s equation- (Nf15 d_XRD_: 7.6 nm and Nf35 d_XRD_: 8.1 nm) is considerably smaller than the corresponding d_TEM_ owing to their multicore nature^29,41,42^. This is supported by higher resolution TEM images where multi-grain constituents of the Nfs are observable while maintaining homogenous crystalline orientation (Supplementary Fig. S1B). In the FT-IR spectrum (Fig. 3A), the peak at 550 cm^−1^ corresponds to the υ(Fe–O). DLS measurements (Table 1 and supplementary Fig. S6) of the as-synthesized Nf15 and Nf35 showed a hydrodynamic diameter by intensity (d_H_) of 30.03 nm (0.366 PdI) and 61.66 nm (0.215 PdI) with a zeta potential of (+)40.7 mV and (+)37.1 mV, respectively. In colloidal systems, d_H_ appears augmented compared to d_TEM_ due to aggregation effects or molecular interactions between the dispersant—in our case water—and the surface of the nanoparticles^43–45^. Nf15 showed some aggregation demonstrated by the increased PdI value and the appearance of a second peak in the size distribution graph (Supplementary Fig. S6), whereas Nf35 appeared with a slight increase in the diameter justified by larger hydration shell volume from the adsorbed water molecules^43,45^.

**Table 1:** DLS data from all nanoformulations; hydrodynamic diameter (d_H_), polydispersity index (PdI) and zeta potential (ζ).

	Nf15	Nf35	cit-Nf15	cit-Nf35	RNf15	RNf35	Rcit-Nf15	Rcit-Nf35	cit-Nf15 @Lipo	cit-Nf35 @Lipo	cit-Nf15 @LipoR	cit-Nf35 @LipoR
Hd (nm)	30.03	61.66	29.81	69.35	114	118.5	27.92	79.81	99.21	92.37	125.1	114.3
PdI	0.366	0.215	0.227	0.243	0.175	0.169	0.192	0.166	0.188	0.24	0.144	0.153
ζ(mV)	40.7	37.1	− 42.2	− 36	− 20.6	− 21.1	− 22.4	− 28.1	− 22.6	− 44.7	− 30.1	− 31

**Figure 2:**
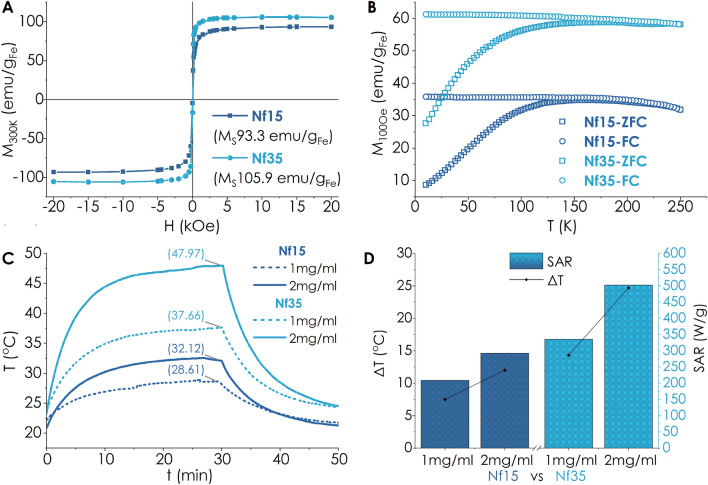
Nf35 shows superior magnetic properties towards magnetic hyperthermia compared with Nf15, as demonstrated by the corresponding (**A**) Hysteresis loop, (**B**) ZFC/FC curves, and their (**C**,**D**) Magnetothermal response.

The room temperature isothermal magnetization of both ferrofluids Nf15 and Nf35 studied by SQUID magnetometry lies in the superparamagnetic regime indicated by the absence of hysteresis while demonstrating high magnetization saturation (M_s_) values of 93.3 and 105.9 emu/g_Fe_, respectively (Fig. 2A), below but close to bulk state^46^. Irreversibility temperature (T_irr_) is defined as the bifurcation point between zero field cooled (ZFC) and field cooled (FC) curves, whereas the blocking temperature (T_B_) represents the maximum of the ZFC curve. T_irr_ of both samples are well below room temperature, also pointing to a superparamagnetic regime. The specific values of T_B_ are; 163 K for Nf15 and 181 K for Nf35 (Fig. 2B). The magnetic hyperthermia (MHT) response after the application of an AMF (f = 395 kHz, H = 18.6 kA/m or 233 Oe) testifies to a concentration-dependent behavior for both nanostructures (Fig. 2C), whilst Nf35 displayed superior SAR values and saturation temperature than Nf15 (Fig. 2D) for the same concentrations. The magnetothermal properties of the synthesized Nfs is strongly dependent on their morphology. The distinctive lower values for M_s_, T_b_ and SAR of smaller sized Nf15 compared to Nf35 can be ascribed to the size and shape anisotropy^20,47–49^ factors having a positive contribution for larger particles.

### Functionalization of iron oxide nanoflowers

**Figure 3:**
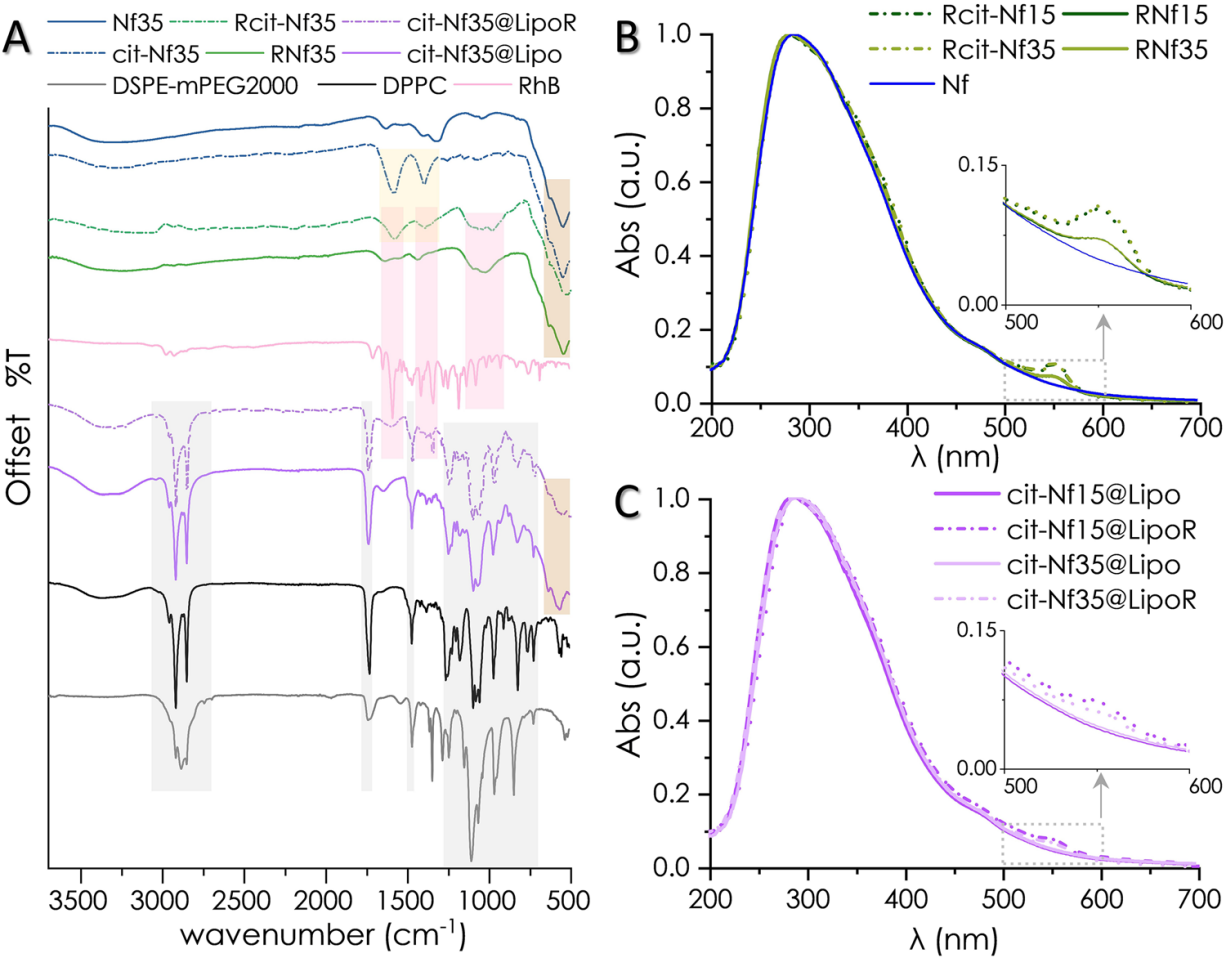
(**A**) Juxtaposed FT-IR spectra of all formulations where highlighted areas indicate the different functionalities (brown: iron oxide, yellow: citrate, pink: RhB, grey: lipids). (**B**,**C**) Observable RhB adsorbed on Nfs or loaded in liposomes in normalized UV–vis spectra at 554 nm.

Trisodium citrate, a common biocompatible functionalization agent, was grafted on the surface of Nfs (cit-Nf15 and cit-Nf35). In the FT-IR spectrum, the appearance (Fig. 3A and supplementary Fig. S2) of intense peaks at 1587 cm^−1^ and 1390 cm^−1^ (υ_as_ + υ_s_ (COO)) supports the presence of chemisorbed carboxylate moieties of citrate radicals^50^. Additionally, the active fluorescent molecule Rhodamine B (RhB) was incorporated on the surface of bare Nfs (RNf15 and RNf35), and citrate functionalized Nfs (Rcit-Nf15 and Rcit-Nf35) by adsorption. The results suggest successful incorporation of RhB onto all four nanostructures preserving their colloidal stability. FT-IR spectra (Fig. 3A, supplementary Fig. S2) reveal characteristic peaks of RhB (1646–1587 cm^−1^ (υ(C=C) in aromatic rings), 1380–1130 cm^−1^ (δ (C–H) in =N + (C_2_H_6_) + υ(C–N)), 1179 cm^−1^ (υ_s_ (C–O–C)), 924 cm^−1^ (δ(OH)) confirming its adsorption on the nanostructures. Uv–vis spectra of RhB modified nanoflowers demonstrate a peak at 554 nm (Fig. 3B) indicative of RhB incorporation in the structure^51^. RhB adsorption was quantified by standard curve method (5.5 μM_RhB_ in RNf15, 5.6 μM_RhB_ in RNf35, 7.6 μM_RhB_ in Rcit-Nf15, 7.9 μM_RhB_ in Rcit-Nf35). Citrate-coated Nfs demonstrated increased adsorption of RhB compared to the uncoated, which is justified by the electrostatic interactions between the negatively charged carboxylate moieties of citrate-already coordinated on the surface of the nanoparticles- and the cationic pendant group [R = N + (C_2_H_6_)] of RhB.

The colloidal stability of the functionalized nanoflowers was assessed via DLS measurements (Table 1 and Fig. S6). After citrate coating, we observed a shift in the zeta potential from highly positive to highly negative, with minimized PdI in cit-Nf15 and cit-Nf35. The apparent increase in d_H_ after citrate and/or RhB functionalization is due to interactions between the carboxylate ions and water molecules or aggregation effects^52^. RhB has a molecular weight almost twice that of trisodium citrate (MW_RhB_: 479.02 vs. MW_cit_: 258.06 g/mol); thus, higher d_H_ was anticipated and confirmed, especially on the bare Nfs coated only with RhB as the absence of citrate as stabilizing agent is essential on the colloidal behavior of the particles.

### Encapsulation in fluorescent thermosensitive liposomes

Based on the localization in vitro data (Fig. 6), negatively charged cit-Nfs demonstrated increased internalization after 24 h of incubation with both cell lines. Thus, we selected cit-Nf15 and cit-Nf35 to encapsulate in thermosensitive liposomes containing RhB and in plain liposomes as control. DPPC (1,2-dipalmitoyl-sn-glycero-3-phosphocholine) is a thermosensitive lipid with a transition temperature (T_m_) at 41.4 °C and is used as a liposome constituent for drug or nanoparticle delivery and release, especially for magnetic nanoparticles to facilitate the effect of MHT^53,54^. DSPE-mPEG_2000_ (N-(Carbonyl-methoxy polyethylene glycol-2000)-1,2-distearoyl-sn-glycero-3-phosphoethanolamine) is a pegylated lipid used in liposome synthesis to increase their stealth in terms of bioavailability^55^. Our liposomes consist of DPPC and DSPEmPEG_2000_ at a ratio of 95:5 mol% and have a hydrodynamic diameter around 100 nm confirmed by DLS (Table 1 and supplementary Fig. S6). RhB (1 mM) was incorporated in the organic lipid phase, whereas Nfs (5 mg/ml) were added during hydration at 60 °C. The increased temperature during hydration and extrusion favors the liquid crystal state of the lipid sheets^56^ thus increasing the ability to engulf cit-Nfs in the final liposomal formulation After 24 h dialysis, the remaining RhB was calculated at 317 μΜ for cit-Nf15@LipoR and 252 μM for cit-Nf35@LipoR according to the UV–vis generated standard curve. FT-IR (Fig. 3A, supplementary Fig. S2) and Uv–vis (Fig. 3C) spectra for all magnetoliposomes confirm the incorporation of Nfs as well as RhB and (Fig. 3B). Although all magnetoliposome nanoformulations were synthesized according to the same procedure, some differences in their colloidal behavior were observed. During the final step of encapsulating cit-Nf35 and cit-Nf15 in plain liposomes through extrusion, we used 100 nm-pore filters and subjected the formulations to dialysis. Size measurement in DLS (Table 1 and supplementary Fig. S6) for cit-Nf35@Lipo exhibited a d_H_ ~ 92 nm, whereas cit-Nf15@Lipo was ~ 100 nm. This was also evidenced in the case of RhB functionalized magnetoliposomes, where we observed d_H_ values of ~ 114 nm for cit-Nf35@LipoR and ~ 125 nm cit-Nf15@LipoR. According to the results the concurrent entrapment of cit-Nfs in thermosensitive liposomes was successful and can be further evaluated for their theranostic potential in comparison with the plain and functionalized Nfs.

### Hemocompatibility assessment

Nanoparticles destined for in vivo biological applications can easily enter systemic circulation and interact with blood components before they reach their target, especially if the desired route of administration is intravenous^57^. Several nanomaterials have been reported to cause hemolysis or disruption of red blood cells’ (RBCs) morphology, the extent of which has been linked to size as well as shape and composition effects^58,59^. Therefore, clinical application of bionanomaterials is dependent on their hemocompatibility.

**Figure 4:**
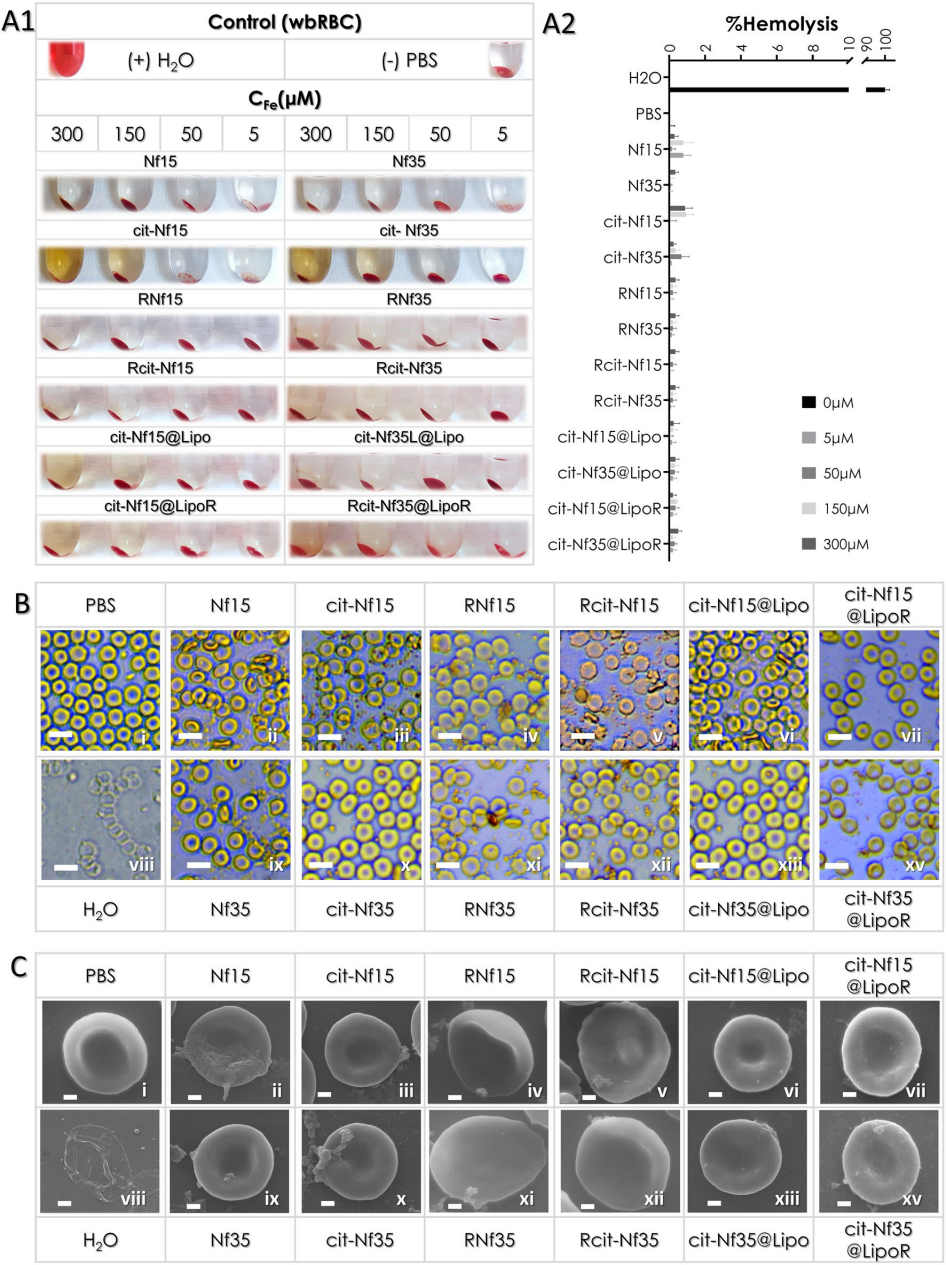
Whole blood RBCs: (**A1**) Optical assessment of hemolysis and (**Α2**) hemoglobin percentage present in each supernatant after 3 h incubation with the samples at different concentrations as measured in ELIZA, (**B**) Optical microscopy (scale bars at 10 μm) and (**C**) SEM images (scale bars at 1 μm) of RBCs after 3 h incubation with the samples at a selected concentration (150 μΜ) on coverslips in 24-well plates.

Herein, all nanostructures were evaluated for their hemocompatibility by calculating the hemolytic effect and the structural changes on RBCs from whole blood (wbRBCs) or isolated (iRBCs). For the hemolysis assay, serial dilutions of the samples were prepared in PBS (5–300 μΜ) and incubated with RBCs for 3 h. All samples exhibited minimal hemoglobin release from the RBCs (< 1%), indicating negligible hemolysis (Fig. 4A, supplementary Fig. S3A) according to the < 10% acceptance limit for biopharmaceuticals^60^. Water and PBS were used as positive and negative hemolysis controls, respectively, illustrated as 100% and 0% hemolyzed control samples. Fluctuations in the measurements are in the error range of the instrument.

Structural interactions of the samples at 150 μΜ_Fe_ and controls with wbRBCs and iRBCs were studied live after 3 h incubation in PBS under an optical microscope (Fig. 4B and supplementary Fig. S3B). After fixation, the same samples were analyzed with SEM for detailed observation (Fig. 4C and supplementary Fig. S3C). Positive control of fully hemolyzed RBCs incubated in water (Fig. 4B,Cviii) appears colorless and deflated. In contrast, the negative control treated with PBS (Fig. 4B,Ci) appears well-rounded and uniform. Citrate coated and liposomal encapsulated Nfs were stable in PBS and did not affect the RBCs morphologically despite attaching on their surface (Fig. 4B,Ciii,x,vi,xiii). Bare Nfs didn’t cause any morphological transformations to the RBCs (Fig. 4B,Cii,ix), but they aggregated and precipitated easily in PBS, also observed by the clear color of the supernatants during the hemolysis assay. Some aggregation was observed in the RhB coated Nfs and cit-Nfs. Aggregation is not an acceptable factor for biological applications. RNf15, R-citNf15 and RNf35 caused deformation of RBCs (Fig. 4B,Civ,v,xi) that may be associated with the presence of RhB leading to different surface interactions with the erythrocytes’ membrane than the non-RhB functionalized samples. Interestingly, this is not the case for Rcit-Nf35 as no aggregation or membrane deformation was observed (Fig. 4B,Cxiii).

The results suggest that all nanoformulations are non-hemolytic, but some of them may cause deformations to the RBC’s morphology. Seemingly, RhB modified bare Nfs of both sizes caused some morphological alterations. Citrate coated Nfs modified with RhB had a contradicting effect between the treated samples of RBCs. While Rcit-Nf15 seems to have caused some deformation on the RBCs, Rcit-Nf35 did not have this effect leaving their morphology intact. This observation can be an indication of size effects; smaller sized nanoparticles are more prone to cause morphological alterations on the RBC membrane^61^. Rcit-Nf35 maintains the highly negative zeta potential compared to the other three Rhodamine B modified nanostructures, thus we can assess that this behavior depends not only on size but also because of more effective repulsive interactions^58^.

### Cytocompatibility and localization in lung cancer cell lines

**Figure 5:**
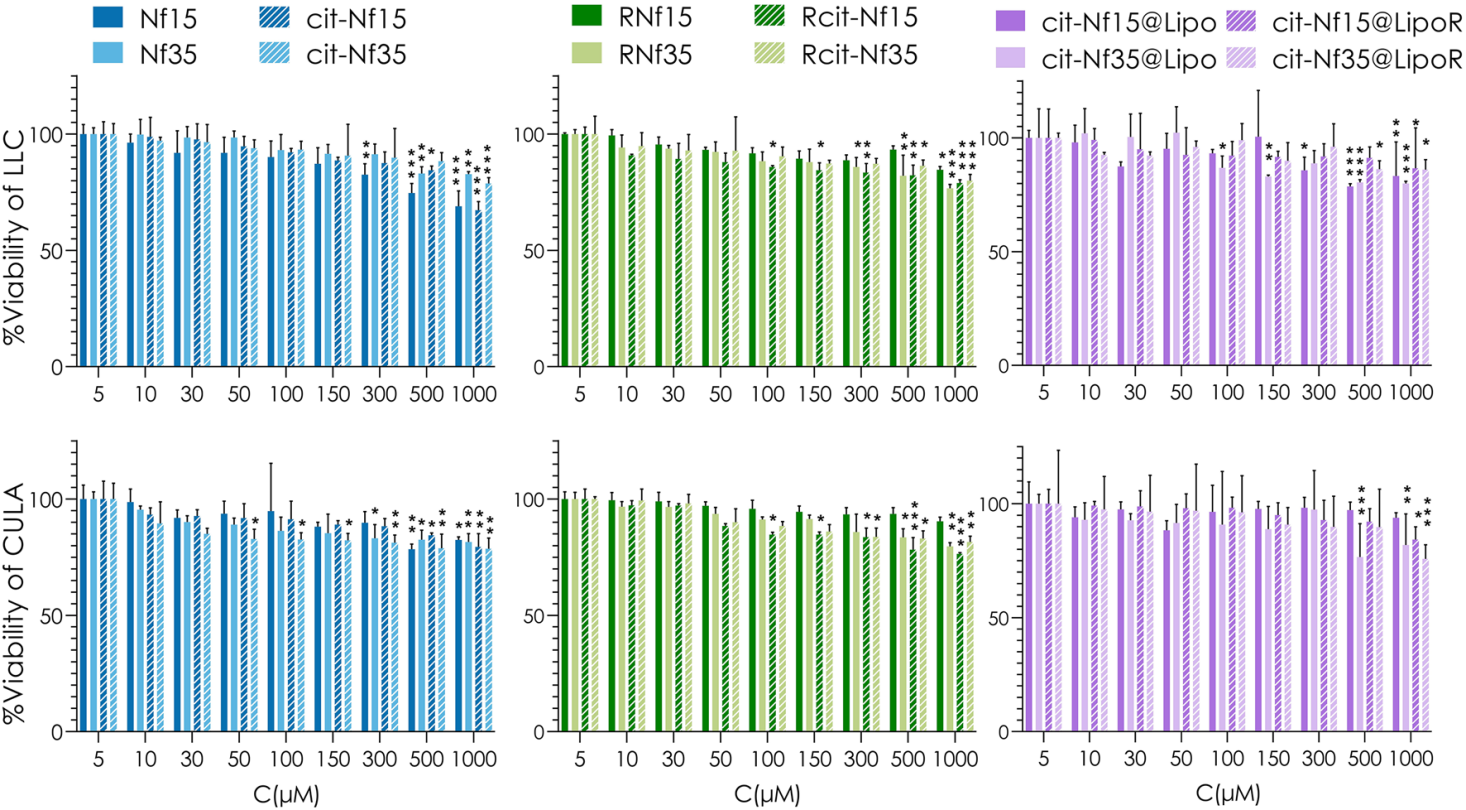
MTT assay results illustrating %Viability of LLC and CULA lung cancer cells after 24 h incubation with each formulation at different iron concentrations (Statistical analysis by two-way ANOVA test with Dunnett’s multiple comparisons test, *0.01 < p < 0.05, **0.001 < p < 0.01, ***0.0001 < p < 0.001).

We chose two lung cancer cell lines, LLC and CULA, to test our samples for cytotoxicity (Fig. 5) at a wide range of concentrations (5–1000 μM_Fe_) commonly encountered in literature^11,62^. According to a two-way ANOVA test followed by Dunnett’s multiple comparisons test, there is a negligible difference between control untreated cells (100% viability) and those treated with each nanostructure up to 300 μΜ_Fe_, whereas between 500 and 1000 μΜ_Fe_ most samples exhibit significant reduction in viability. Hence, there is a linear regression for increasing concentrations of each sample, with the lowest value appearing for Nf15 and cit-Nf15 at ~ 70% at the highest concentration in LLC cell line, whereas in CULA cells the corresponding values are at ~ 80%. According to a second two-way ANOVA test followed by Tukey’s multiple comparisons among the different types of samples, no notable differences were observed for the same concentrations, except slightly significant decrease in viability of LLC treated with 1000 μΜ_Fe_ Nf15 and cit-Nf15 when compared with liposomal encapsulated samples. Bare or citrate coated Nf15 compared to Nf35 and cit-Nf35 showed 10% less viability at the highest concentrations in LLC. This pattern could be an indication that smaller sized nanoparticles are indeed more prone to cause toxic effects, while the addition of a lipid bilayer offers a stealth property to them, which is in accordance with literature^63^. Between the two cell lines, LLC cells showed a slight insignificant decrease in viability compared to CULA cells, which seem to be more resistant to the different sample treatments at higher concentrations.

**Figure 6:**
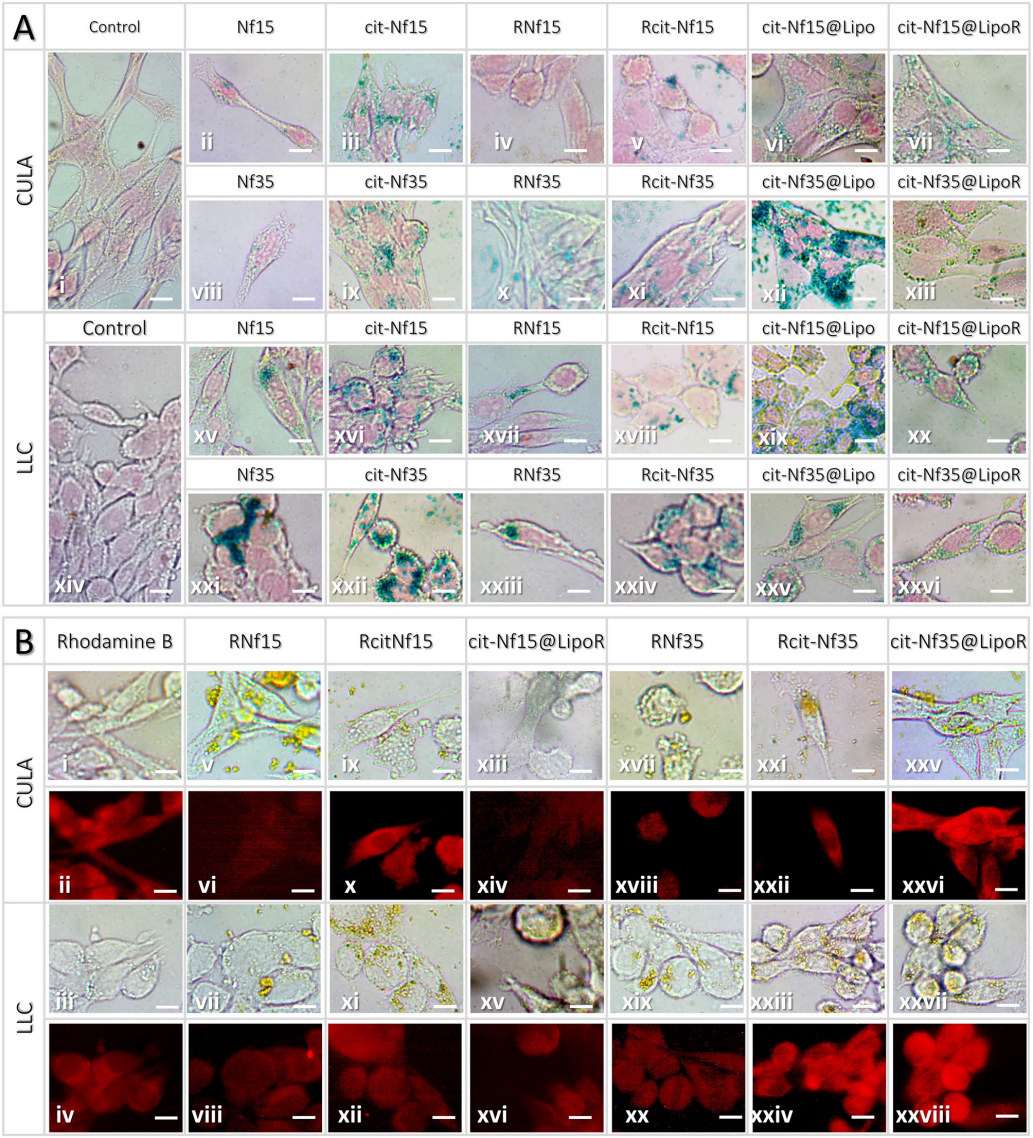
(**A**) Optical microscopy images of all Nf formulations stained with Prussian blue (**B**) Fluorescent microscopy images of RhB modified samples. Both sets of images were acquired after 24 h treatment and incubation in either CULA or LLC cell lines. (All scale bars at 10 μm).

To explore the route of intracellular localization of our formulations, we followed two different in vitro imaging protocols; observation of magnetic nanoparticles via Prussian blue staining under optical microscopy (Fig. 6A) and fluorescent microscopy for the RhB modified formulations (Fig. 6B). Prussian blue staining revealed differences in cellular internalization depending on the size and the surface modification of the samples. Smaller-sized Nf15 with or without functionalization had an overall observable profile of decreased endocytosis compared to Nf35. Citrate-coated Nfs (Fig. 6Aiii,ix,xvi,xxii) exhibited increased localization in the cytoplasm in both cell lines, compared to bare Nfs (Fig. 6Aii,viii,xv,xxi). Rhodamine modified Nf15 with or without citrate (Fig. 6A iv-v, xvii-xviii) appeared to be rather aggregating than internalizing, whereas the corresponding functionalized Nf35 (Fig. 6Ax–xi,xxiii–xxiv) were observed in the cytoplasm, especially Rcit-Nf35. Liposomal encapsulated cit-Nfs (Fig. 6Avi,xii,xix,xxv) showed increased internalization compared to plain cit-Nfs, with a slight decrease in the RhB modified magnetoliposomes (Fig. 6Avii,xiii,xx,xxvi) but still higher than the unencapsulated ones. This was further verified by fluorescent microscopy and quantification of the fluorescent signal with respect to the background (Supplementary Fig. S7). Rcit-Nfs (Fig. 6Bix–xii,xxi–xxiv) showed more intense signal-originating from the cytoplasm-than RNfs (Fig. 6Bv–viii,xvii–xx) and even greater for Rcit-Nf35 and cit-Nf35@LipoR (Fig. 6xxi–xxiv,xxv–xxviii). The non-leaching profile of RhB modified samples (Supplementary Fig. S5)-discussed in the next section- validates that the signal originates solely from the internalized particles.

The results from the two different protocols followed to assess the cellular localization of our nanoformulations come into an agreement, as both methods revealed an accumulation of the particles in the cytoplasm. Especially for the Nf35 family of samples we observed increased internalization in the cytoplasm, compared to the Nf15 family of samples, validated by both Prussian blue staining and fluorescent microscopy. Moreover, the observed increase of internalization after liposomal encapsulation can be credited to the enhanced elasticity of the structure and the augmented affinity towards cellular membranes compared to unencapsulated particles^64^.

### Cell death induced by magnetic hyperthermia

**Figure 7:**
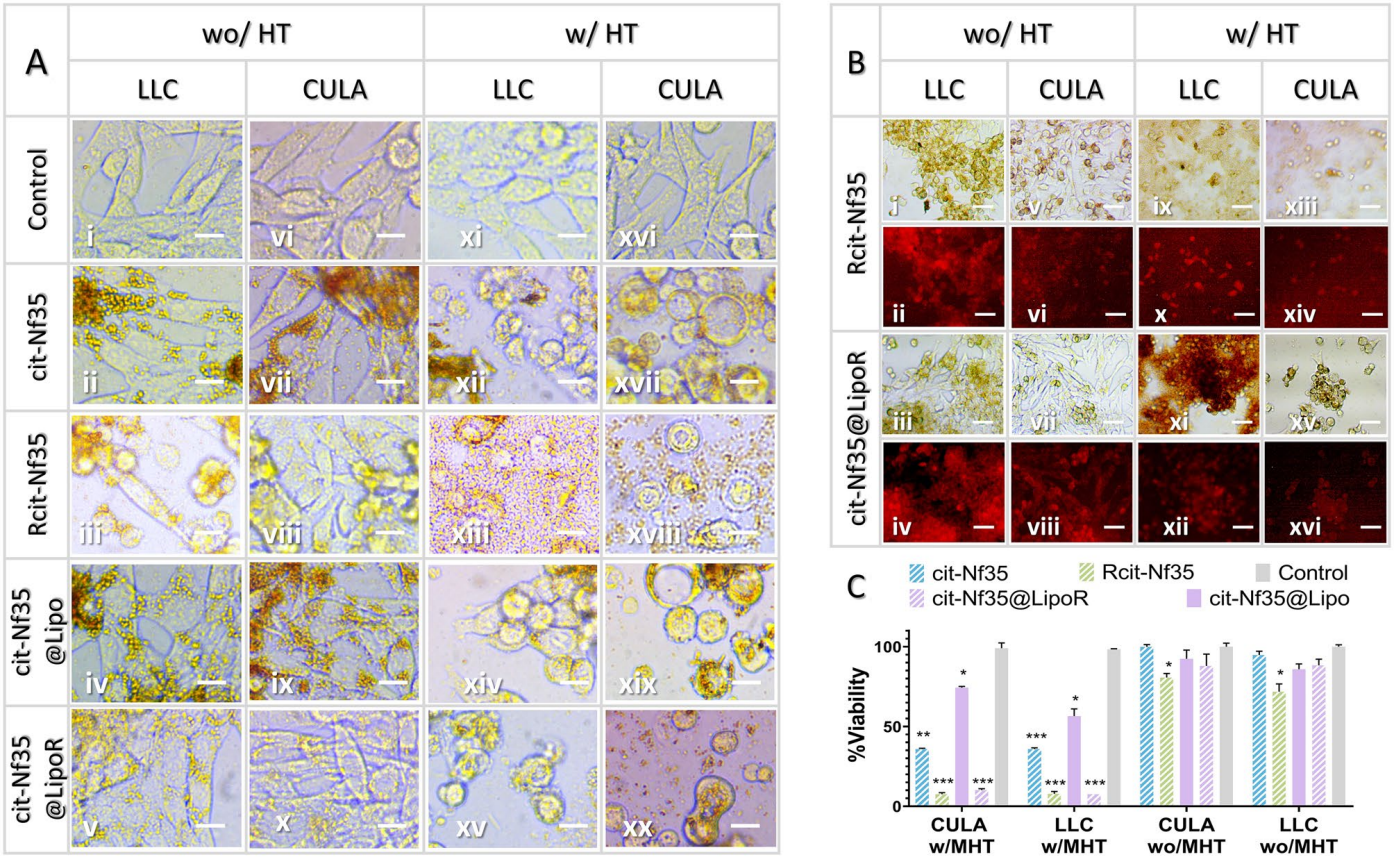
In vitro magnetic hyperthermia after-effects 24 h post treatment (**A**) Live optical microscopy (**B**) Optical fluorescent microscopy (all scale bars at 10 μm) after fixation (**C**) MTT viability assay (*0.01 < p < 0.05, **0.001 < p < 0.01, ***0.0001 < p < 0.001).

To perform in vitro MHT, we chose specific samples based on the findings from their magnetothermal response as well as cytocompatibility and localization assays. The selected samples were cit-Nf35, Rcit-Nf35, cit-Nf35@Lipo, and cit-Nf35@LipoR. Pellets of approximately 1.2–1.5*10^6^ LLC or CULA cells were formed by gentle centrifugation, and 500 μl culture medium containing 27mM_Fe_ (1.5 mg_Fe_/ml) of each sample was introduced. Each sample was subjected to the same conditions; thermostated at 36.6 °C and underwent MHT for 15 min. Control samples were thermostated at 36.6 °C and remained in the incubator for 15 min. According to the thermal curves (Supplementary Fig. S4), Nf35 reached 44 °C faster than the other samples, but most samples reached 42 °C in about 5 min and endured another 10 min of MHT at 43–44 °C. By removing the supernatant immediately after the 15 min treatment and resuspending each pellet in DMEM, we could keep only the amount of the nanoparticles that interacted (internalized or surface-attached) with the cells. Each cell suspension was divided into 3 parts and re-seeded in culture plates for 24 h. One part for live microscopy (Fig. 7A), the second for fluorescent microscopy (Fig. 7B), and the third for MTT (Fig. 7C). The concentration is a crucial factor for MHT experiments both in vitro and in vivo^65^. We chose the specific concentration of 27mM_Fe_, considering their magnetothermal response (Fig. 2C,D) with respect to the viability results (Fig. 5), by scaling up the iron concentration to match the cellular concentration of the pellet. In more detail, the cell pellets, created to simulate a mini-tumor for in vitro MHT, have 50–75 times higher cellular concentration than in the MTT experiment. At concentrations [300–500] μΜ_Fe_ (Fig. 5) the cellular viability remained over 80% in both cell lines, whilst the theoretically equivalent -for higher cellular density- concentration range is^15–37^ mM_Fe_. The selected concentration of 27 mM_Fe_ falls within this range, while retaining the desired magnetothermal response to evaluate the therapeutic effect of our samples after in vitro MHT.

We observed severe cytological alterations in size and shape of both LLC and CULA cells after applying MHT, compared with the control groups which remained intact and proliferated normally (Fig. 7A). Cells appear apoptotic in all samples treated with MHT (Fig. 7Axi–xx), but at different stages. Rhodamine B containing nanostructures after MHT presented extensive cellular deformation (Fig. 7Axii,xv,xvii,xx) indicative of late apoptosis, which is further supported by considerably low viability for Rcit-Nf35 (CULA: 7.6% and LLC: 7.8%) and cit-Nf35@LipoR (CULA: 10.2% and LLC: 7.5%) (Fig. 7C w/MHT). Rcit-Nf35 and cit-Nf35@LipoR control samples without MHT appear in the cytoplasm without affecting the cell structure, evidenced in both optical (Fig. 7Aiii,vii,v,x) and fluorescent (Fig. 7Bi–viii) microscopy. Cells treated with cit-Nf35 after MHT (Fig. 7Axii,xvii) appeared as apoptotic at a lesser extent with notably decreased viability in both cell lines (CULA: 35.9% and LLC: 35.8%) (Fig. 7C). Apoptotic cells were observed co-existing with well-defined live cells in cit-Nf35@Lipo samples undergoing MHT (Fig. 7Axiv,xix), which was reflected by higher cellular proliferation rates (CULA: 74.3% and LLC: 56.5%) (Fig. 7C). The unaffected cellular structure (Fig. 7Ai–x) and substantially increased viability (Fig. 7C wo/MHT) of all unexposed to MHT samples -similarly to the MTT results in Fig. 5 at the range of 300–500 μΜ_Fe_—leads to the assumption that the observable cell death is solely dependent to the contribution of MHT.

After calculating the remaining iron concentration in the cell pellets, we observed that almost half of the amount of the treating nanoformulations actually remains in the cell pellet after the supernatant’s removal. Interestingly, the cell pellets subjected to MHT presented slightly higher remaining iron concentration than the control samples without MHT (Supplementary Table S1). This phenomenon can be attributed to the increased cellular membrane fluidity, after exposure to MHT at 44 °C, resulting in the enhancement of permeability towards the samples^66^.

Rhodamine B containing nanostructures showed significant cell damage and considerable proliferation decrease in both cell lines after MHT exposure. We suspect that the loaded RhB was released from the thermosensitive lipid bilayer because of the temperature increase after MHT, leading to accelerated apoptosis stemming from the magnetothermal response. To explore this possibility, we performed a release experiment for Rcit-Nf35 and cit-Nf35@LipoR in presence and absence of a magnetic field. Indeed, the results (Supplementary Fig. S5A) suggest that 32% of the liposomal encapsulated RhB was released under MHT, whereas Rcit-Nf35 demonstrated minimal release at 3%. In the absence of an AMF, no significant release was observed. The difference in the release profile between the two samples under MHT can be attributed to their structure; in Rcit-Nf35, RhB is stably conjugated through chemical adsorption on the surface of the nanoflowers by means of electrostatic interactions, whereas in cit-Nf35@LipoR, RhB is bounded inside the thermosensitive liposomes through passive loading and can be released with heating. This behavior is also supported by the drop in fluorescent signal intensity after MHT for cit-Nf35@LipoR (Supplementary Fig. S8), in which case the released RhB is removed along with the supernatant. Hence, concurrent in vitro MHT and RhB interaction with the cells leads to significant decrease in viability. In the absence of an AMF Rcit-Nf35 presented ~ 10% decreased viability compared to the RhB containing magnetoliposomes, probably stemming from the immediate exposure to the grafted RhB. Cells treated with cit-Nf35 and cit-Nf35@Lipo overall exhibit higher cellular viabilities with or without MHT, compared to the ones that contain RhB. In the context of Rhodamine B having been reported to cause DNA damage and oxidative stress^67–69^, we can conclude that it contributes synergistically to MHT induced cancer cell apoptosis, in our experimental set-up.

## Conclusion

We successfully synthesized iron oxide nanoflowers of two sizes − 15 and 35 nm-exhibiting size-dependent magnetothermal response, with Nf35 being a more effective nanoheater. Citrate was grafted on the surface of the particles improving their colloidal behavior. Rhodamine Β was adsorbed effectively on their surface but proved to impose slight aggregation effects especially in the absence of citrate. Cit-Nf15 and cit-Nf35 were encapsulated successfully in thermosensitive liposomes where Rhodamine was incorporated in the lipid bilayer, minimizing any possibility of aggregation. None of the formulations induced hemolysis or significant disturbances in the RBC morphology. Nfs, localize in the cytoplasm of the cells in different amounts depending on their size and coating. Cytotoxicity profile for all formulations in LLC and CULA lung cancer cell lines showed negligible effect without MHT, but after exposure to an AMF, the viability of the cells substantially dropped. Rhodamine modified liposomes containing 35 nm iron oxide nanoflowers (cit-Nf35@LipoR) proved to be a promising candidate for MHT and cellular imaging.

## Methods

### Synthesis of iron oxide nanoflowers (Nfs)

Iron oxide nanoflowers were synthesized via a modified polyol method^35^. In a three-neck round, bottom flask 1.082 g (4 mmol) FeCl_3_.6H_2_O and 0.4 g FeCl_2_.4H_2_O (2 mmol) was dispersed in 80 g of DEG and NMDEA mixture at a 1:1 v/v ratio. Separately, 0.64 g (16 mmol) NaOH were dispersed in 40 g of 1:1 v/v mixture. After 1 h, the NaOH dispersion was added to the mixture of iron salts and left under stirring for 3 h. The temperature was increased at a heating rate of 2 °C/min until it reached 210 °C, where it was left for 30 min (Nf15) or 1 h (Nf35). When the reaction mixture cooled down to room temperature, the Nfs were magnetically separated with a neodymium magnet. The precipitate was washed thrice with a mixture of ethanol and ethyl acetate (1:1 v/v), twice with 10% HNO_3_, twice with ethanol, and once with diethyl ether. The isolated Nfs were redispersed in water, creating a stable ferrofluid, which was then oxidized by adding 8.3 g Fe(NO_3_)_3_·9H_2_O under stirring at 80 °C for 45 min. The oxidized Nfs were magnetically separated, washed twice with 10% HNO_3_, twice with ethanol, and once with diethyl ether. The produced maghemite Nfs were dispersed in water and stored at 4 °C.

### Coating Nfs with citrate ions (cit-Nfs)

Half portion of Nf15 or Nf35 were subsequently modified with citrate ions. Briefly, we added 0.8 mol C_6_H_5_Na_3_O_7_.2H_2_O per 1 mol of iron content of Nf colloidal solution in a 50 ml round bottom flask under stirring for 30 min at 70 °C. The modified products (cit-Nf15 and cit-Nf35) were magnetically separated, washed once with acetone, twice with ethanol, and redispersed in water.

### Functionalization of Nfs and cit-Nfs with Rhodamine B (RNfs and Rcit-Nfs)

The functionalization of Nfs with RhB is based on adsorption phenomena^70^. Nf and cit-Nf products were dispersed in a solution of RhB at a 2:1 molar ratio and left under vigorous agitation overnight. Each sample was purified via magnetic separation and redispersion in water several times. Rhodamine B quantification was conducted via standard curve method. The final products were redispersed in water.

### Liposomal co-encapsulation of RhB and cit-Nfs

To prepare plain magnetoliposomes (cit-Nf@Lipo), DPPC and DSPE-mPEG were mixed (95:5) in a 10 ml conical flask at a final total lipid concentration of 24 mM in 1 ml chloroform. After vigorous agitation for 5 min, the mixture was dried with a rotary evaporator for approximately 1 h at room temperature and transferred under N_2 (g)_ flow for another hour to create a thin lipid film. 1 ml aqueous solution of Nfs at a concentration of 5 mg_Fe_/ml was then added to the film for hydration, and the flask was placed in a water bath at 60 °C for 10 min under agitation. Following, 10 alternating cycles of freezing in liquid N_2_ and thawing at 60 °C were performed, then 1 min sonication, and the mixture was left for 5 min in tranquility in the water bath. The final mixture was transferred in a gas-tight syringe followed by 20 consecutive extrusions via 200 nm and another 20 via 100 nm porous polycarbonate film in a preheated (60 °C) Avanti® extruder set. The same steps were followed for the co-encapsulation of Nfs and RhB (cit-Nf@LipoR), where this time RhB was introduced in the primary chloroform lipid mixture at a final concentration of 1 mM. To purify the final product, it underwent dialysis for 24 h at 10 °C against 250 ml water. During this step, low temperature was ensured to avoid liposomal destruction and leaching of encapsulated RhB or Nfs, while the water was changed five times.

### Determination of iron concentration

The final concentration of all products, expressed as a total iron concentration, was determined by the colorimetric phenanthroline assay. The phenanthroline protocol is based on the affinity of iron to form complexes with 1,10-phenanthroline having characteristic red color and UV–vis absorption at 510 nm. Briefly, the ferrofluid is digested in HCl (1:1 v/v) at 70 °C for 30 min. The sample is then diluted with water, and 200 μL of it are mixed with 50 μL HONH_2_·HCl (10 mg/ml) and 450μL CH_3_COONa (125 mg/ml). Finally, 300 μL of 1,10-phenanthroline monohydrate is added to acquire the characteristic red complex. The same procedure was followed to create a standard curve from a stock solution of FeCl_2_.4H_2_O in various known concentrations expressed as “mg_Fe_/ml” for most characterization techniques, while for biological assays, it’s expressed in “μΜ_Fe_”. The samples were measured in UV–vis and their absorption at 510 nm.

### Electron microscopy

Scanning electron microscopy (SEM) images were obtained on a FEI Inspect microscope with Tungsten filament, operating at 25 kV. Transmission electron microscopy (TEM) images were obtained on FEI CM20 microscope operating at 200 kV, equipped with a Gatan GIF200 Energy Filter utilized for EF-TEM elemental mapping.

### X-ray diffraction (XRD)

Crystal structure of freeze-dried powders was determined by X-ray diffraction performed in an Agilent Technologies SuperNova diffractometer with a Mo micro-focused source (Kα2 = 0,713,067). Crystallite size was determined by Scherrer’s formula after Lorentzian peak fitting on the (311) diffraction line.

### Dynamic light scattering (DLS)

The hydrodynamic diameter (H_d_) and zeta potential were determined by DLS spectroscopy with a Malvern Instruments Zetasizer Nano Series; the measurements were performed in triplicates with 10 runs each at 0.055 mg_Fe_/ml ≡ 1 mM_Fe_. The presented graphs correspond to the averaged results.

### Fourier transform infrared (FT-IR) Spectroscopy

FT-IR spectra were obtained by a Perkin Elmer Spectrum 100 Spectrometer. The samples were dried under vacuum and scanned 4 times over the range of 4000–380 cm^−1^.

### Ultraviolet–visible (UV–vis) spectroscopy

UV–vis absorption spectra were obtained on a Jasco V-650 spectrometer at a range of 190–900 cm^−1^.

### Superconducting quantum interference device (SQUID) magnetometry

Magnetic measurements were done on the SQUID-based commercial magnetometer Quantum Design MPMS XL5, on the ferrofluid samples, sealed in thermostable plastic containers at 6.2 mg_Fe_/ml concentration. Temperature dependence of magnetization was measured in the range of 10–250 K at the applied DC field of 100 Oe. In the zero-field cooled (ZFC) protocol, samples were cooled down without field, and magnetization was recorded during the reheating in the applied field of 100 Oe, under the same temperature rate of 1 K/min. Field cooled (FC) protocol followed the same pattern, except the sample was cooled down in the applied field of the same strength. Isothermal magnetization at room temperature (300 K) was recorded in the field range of ± 20 kOe.

### Magnetic hyperthermia (MHT)

Magnetic hyperthermia experiments were conducted in an Ambrel non-adiabatic system equipped with a 3-turn coil operating frequency of 395 kHz and a magnetic field of 18.6 kA/m. The temperature rise of the samples was recorded via a fiber-optic thermometer in an aqueous solution within glass vials. Specific absorption rates (SAR) values were calculated according to the equation

1 $$SAR=\frac{{C}_{H2O}{m}_{H2O}}{{m}_{Fe}}\left(\frac{dT}{dt}\right)$$

where C_H2O_ = 4185 J/(kg.K), m_H2O_ = 1 g, m_Fe_ = 1 or 2 mg and dT/dt is calculated from the hyperthermia curve on the linear part of the first 20 s.

### Hemocompatibility study

The hemolytic and morphological alterations of red blood cells (RBCs) of all the samples were studied on clinical discarded blood samples. The experiment was conducted either in whole blood (wbRBCs) or isolated RBCs (iRBCs). Isolation of the RBCs took place by gravity gradient using Histopaque and centrifuging at 480 g, followed by consecutive dilutions and centrifugations with 10 mM PBS until a clear supernatant was acquired. iRBCs were diluted with PBS at their initial concentration in WB.

### Hemolysis assay

The hemolysis assay was conducted by adding 15 μl of wbRBC or iRBC in 500 μl of each sample (300, 150, 50, 5 μΜ_Fe_ ≡ 16.8, 8.4, 2.8, 0.3 μg_Fe_/ml) in a sterile Eppendorf tube and incubating at 37 °C for 3 h. PBS was used as negative and water as positive control. Samples were centrifuged for 10 min while supernatants were removed and measured in triplicates in an Eliza plate reader at 540 nm. To remove the interfering absorption of the iron from the assay for each sample, a control experiment was conducted where they were incubated at 37 °C for 3 h in plain PBS, and the supernatant at each concentration after centrifugation was used as a control. The hemolysis ratio was calculated by the equation:

2 $$\frac{{A}_{sample}-{A}_{PBS}}{{A}_{H2O}-{A}_{PBS}}*100$$

### RBC Morphology study

In a 24-well plate containing sterile cover glasses, 10^4^ iRBC or wbRBC were seeded and treated with each sample at the same concentration of 150 μΜ_Fe_ ≡ 8.4 μg_Fe_/ml. The cells were incubated for 3 h and studied live under an optical microscope. Finally, the supernatant was removed, the cells were fixated with 2.5% glutaraldehyde in PBS followed by dehydration via consecutive washings with ethanol solutions (30–50–70–80–90–95–100% v/v) every 10 min. The cover glasses were then removed, placed on a carbon tapped SEM holder, dried under ambient conditions, and coated with a thin layer of gold. The fixation procedure was followed to observe the RBC with SEM.

### Cell culture of lung cancer cell line

Lewis lung adenocarcinoma (LLC) and urethane induced lung adenocarcinoma (CULA) cell lines were kindly provided by Dr. Maria Tsoumakidou Lab from the Institute of Bioinnovation Biomedical Sciences Research Center “Alexander Fleming”. The medium used for cell culturing was glutamine-rich DMEM (Dulbecco’s Modified Eagles Medium, without Ca or Mg) supplemented with 10% FBS and 1% penicillin/streptomycin as antibiotics. The incubation conditions were; 37 °C with 5% CO_2_ flow.

### MTT cell viability assay

The assessment of cellular viability was performed through the -well established in literature- MTT assay. Briefly, LLC or CULA lung cancer cell lines were seeded in 96-well plates at a cell density of 1*10^4^ cells per well and incubated for 24 h (final cellular concentration at 2–2.5 10^4^ cells/well). The culture medium was then removed and replaced by culture medium containing different concentrations of each sample in triplicates (1000, 500, 300, 150, 100, 50, 30, 10, 5 μΜ_Fe_ ≡ 55.8, 27.9, 16.8, 8.4, 5.6, 2.8, 1.7, 0.6, 0.3 μg_Fe_/ml). After a 24 h incubation, the medium was removed, washed with PBS, and treated with 1 mg/ml MTT solution (3-(4,5-dimethylthiazol-2-yl)-2,5-diphenyltetrazolium bromide) in PBS for 4 h. The purple formazan crystals formed were diluted in 100 μl of 2-propanol, and the absorbance was measured with an Eliza plate reader at 540 nm with a reference at 620 nm. Similarly, MTT assay was performed on the samples involved in the in vitro* ΜΗΤ.* Presented data are average ± standard deviation (SD) of n = 3. Statistical analysis was performed using (a) two-way ANOVA followed by Dunnett's multiple comparisons of each sample versus control test and (b) two-way ANOVA followed by Tukey's multiple comparisons between the different samples at each concentration. Both tests were conducted at 90% confidence interval with *0.01 < p < 0.05, **0.001 < p < 0.01, ***0.0001 < p < 0.001.

### Prussian blue staining and optical microscopy

The visualization of nanoparticle uptake by LLC or CULA cells was achieved by the Prussian Blue staining method. Cells were seeded in 6-well plates containing sterile coverslips at a cell density of 1*10^5^ cells per well and incubated for 24 h. The culture medium was then removed and replaced by culture medium containing each sample at a concentration of 150 μM_Fe_. After 24 h of incubation, the medium was removed, the cells were washed with PBS and fixated with 4% paraformaldehyde in PBS for 20 min at 37 °C. The fixated cells were washed twice with PBS, incubated with Pearls solution (1:1 solution of 4% w/v K_4_Fe(CN)_6_.3H_2_O in PBS and 4 M HCl in PBS) for 30 min and washed again with PBS. Nuclear fast red 0.02% solution was added for 5 min and quickly washed with PBS. The coverslips were removed and placed on glass slides to be studied with optical microscopy.

### Widefield optical fluorescence microscopy

The uptake of samples containing RhB was comparatively studied by fluorescent excitation, taking advantage of their innate fluorescence without PB staining. The apparatus employed for the experiments was OMAX Trinocular Compound EPI-Fluorescence Microscope M834FLR with 1.3MP CMOS Camera (Blue filters: Excitation 410–490 nm, Emission: 515 nm—Green filters: Excitation 490–540 nm, Emission: 590 nm). Because of the high nanoparticle content in the samples after in vitro magnetic hyperthermia, the noise from the fluorescent signal was high; thus, we were able to acquire only lower magnification photos from the microscope. The fluorescence intensity, of selected fluorescent regions of interest (ROIs) in the acquired images, was measured through the ImageJ software, with respect to the background and the measured area, as an expression of the relative mean signal intensity.

### In vitro magnetic hyperthermia

The samples' cellular uptake after applying magnetic hyperthermia for 30 min was evaluated in LLC and CULA cell lines. Cells were seeded in 6-well plates at a cell density of 3*10^5^ cells per well and incubated for 24 h to confluency (1.2–1.5*10^6^ cells per well). The culture medium was removed, the cells from each well were trypsinized and pelleted in sterile Eppendorf vials. In each vial containing the cell pellet, we added 500 μl culture medium containing 1.5 mg_Fe_/ml ≡ 27 mM_Fe_ cit-Nf35, Rcit-Nf35, cit-Nf35@Lipo, cit-Nf35@LipoR, and a blank with pure culture medium, in duplicates. The first series was subjected to magnetic hyperthermia for 10 min at 43–44 °C, whereas the second was used as control and incubated at 37 °C for the same time. The same AMF as in plain MHT was applied; 395 kHz and 18.6 kA/m. The supernatants were removed, each pellet was diluted and dispersed in fresh medium. A portion of each sample was then seeded in 6-well plates to facilitate optical observation and another in 96-well plates (in triplicates) to perform MTT assay. Both evaluation protocols were conducted following a 24 h incubation post-treatment. The supernatants were collected in order to determine -by subtraction- the percentage of internalized iron through the phenanthroline assay.

### Rhodamine B release experiment under magnetic hyperthermia

The experiment was performed to simulate the conditions of in vitro magnetic hyperthermia and assess the potential of hyperthermia inducing the release of RhB from Rcit-Nf35 and cit-Nf35@LipoR. 500 μl of each sample at 1.5 mg_Fe_/ml, was dialyzed against thermostated water at 37 °C in a custom-made dialyzer (Supplementary Fig. S5B), with or without the application of an AMF (f = 395 kHz and H = 18.6 kA/m) for 15 min. The absorbance of the samples was measured at 554 nm with UV–vis spectroscopy and the released amount of RhB was determined through the equation:

3 $$\%Release=\frac{{m}_{RhBi}-{m}_{RhBf}}{{m}_{RhBi}}*100= \frac{{released m}_{RhB}}{{m}_{RhBi}}*100$$

where m_RhBi_ is the mass of RhB in the sample initialy and m_RhBf_ is the mass of RhB in the sample after the release experiment.

### Statements

All methods were carried out in accordance with relevant guidelines and regulations. All experimental protocols do not need to be approved by the institute. The informed consent was obtained from all subjects and/or their legal guardian(s). All experiments conducted with cell cultures and discarded blood from unknown patient approval is not necessary.

## Supplementary Information

Supplementary Information.
