# Supplementary material for: Iron oxide nanoflowers encapsulated in thermosensitive fluorescent liposomes for hyperthermia treatment of lung adenocarcinoma
Source: Sci Rep. 2022 May 24;12:8697. doi: 10.1038/s41598-022-12687-3 (PMC9130318; doi:10.1038/s41598-022-12687-3)
Supplement: Supplementary file 1 — Supplementary Information. [file 41598_2022_12687_MOESM1_ESM.docx]

Supporting Information:

Iron Oxide Nanoflowers Encapsulated in Thermosensitive Fluorescent Liposomes for Hyperthermia Treatment of Lung Adenocarcinoma

Maria Theodosiou^1,2^, Elias Sakellis^2^, Nikos Boukos^2^, Vladan Kusigerski^3^, Beata-Kalska Szotko^4^, Eleni Efthimiadou*^1,2^

^1^Laboratory of Inorganic Chemistry, Department of Chemistry, National and Kapodistrian University of Athens, Greece. ^2^ Institute of Nanoscience and Nanotechnology, National Center for Scientific Research “Demokritos”, Greece. ^3^Institute of Nuclear Sciences Vinca, University of Belgrade, Republic of Serbia. ^4^ Faculty of Chemistry, University of Białystok, Poland.


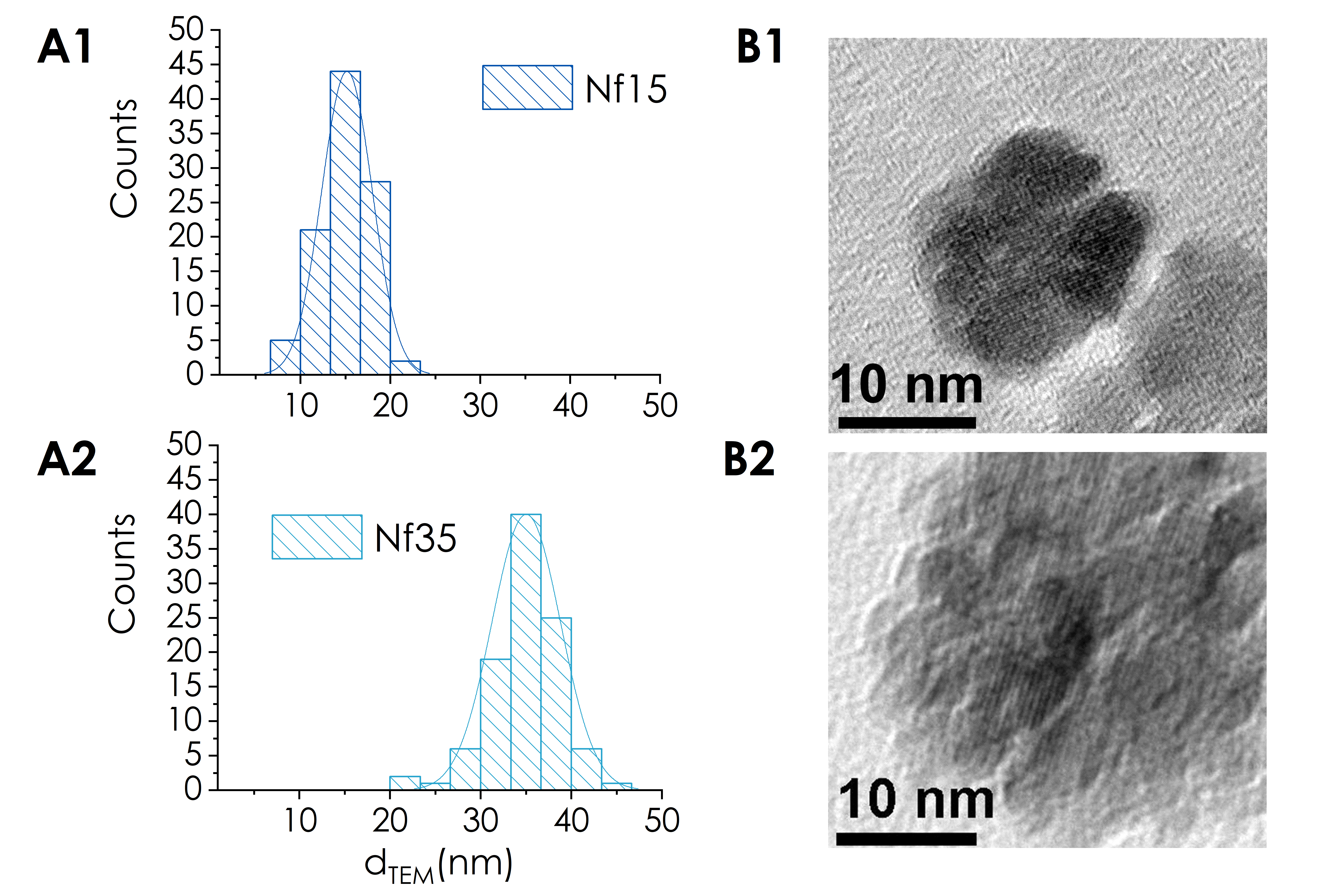


Figure S 1: A1-2) Lognormal distribution of 100 particles’ diameter and high resolution TEM (right) of B1) Nf15 and B2) Nf35.


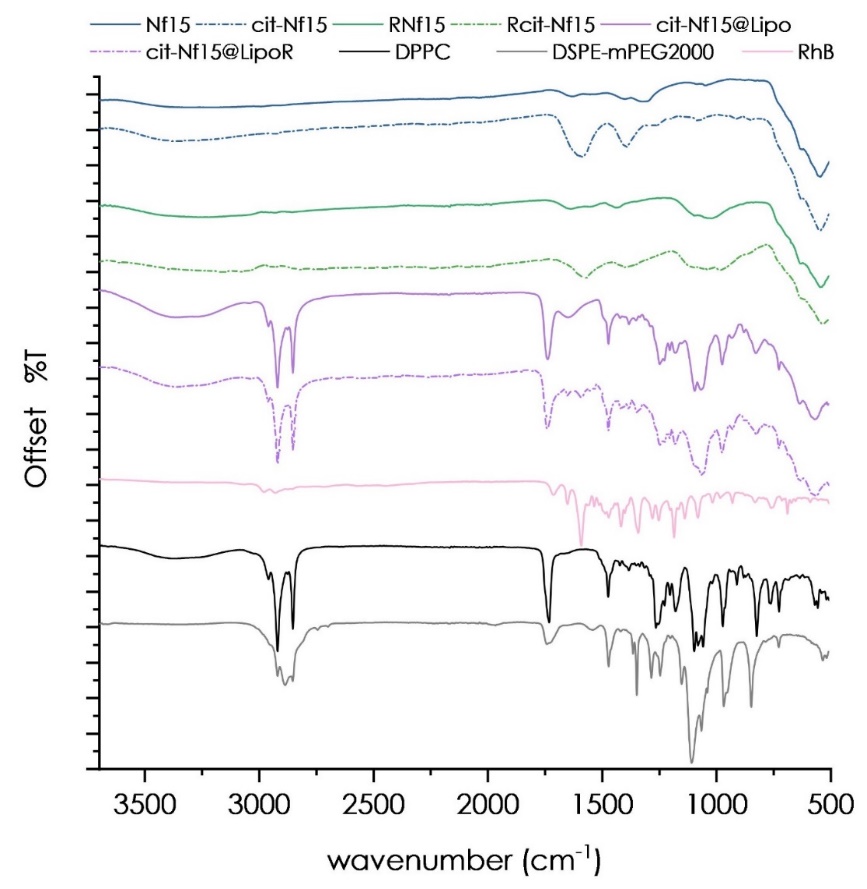


Figure S 2: FT-IR spectra of Nf15 containing nanoformulations in comparison with the materials used for coating and encapsulation in liposomes.


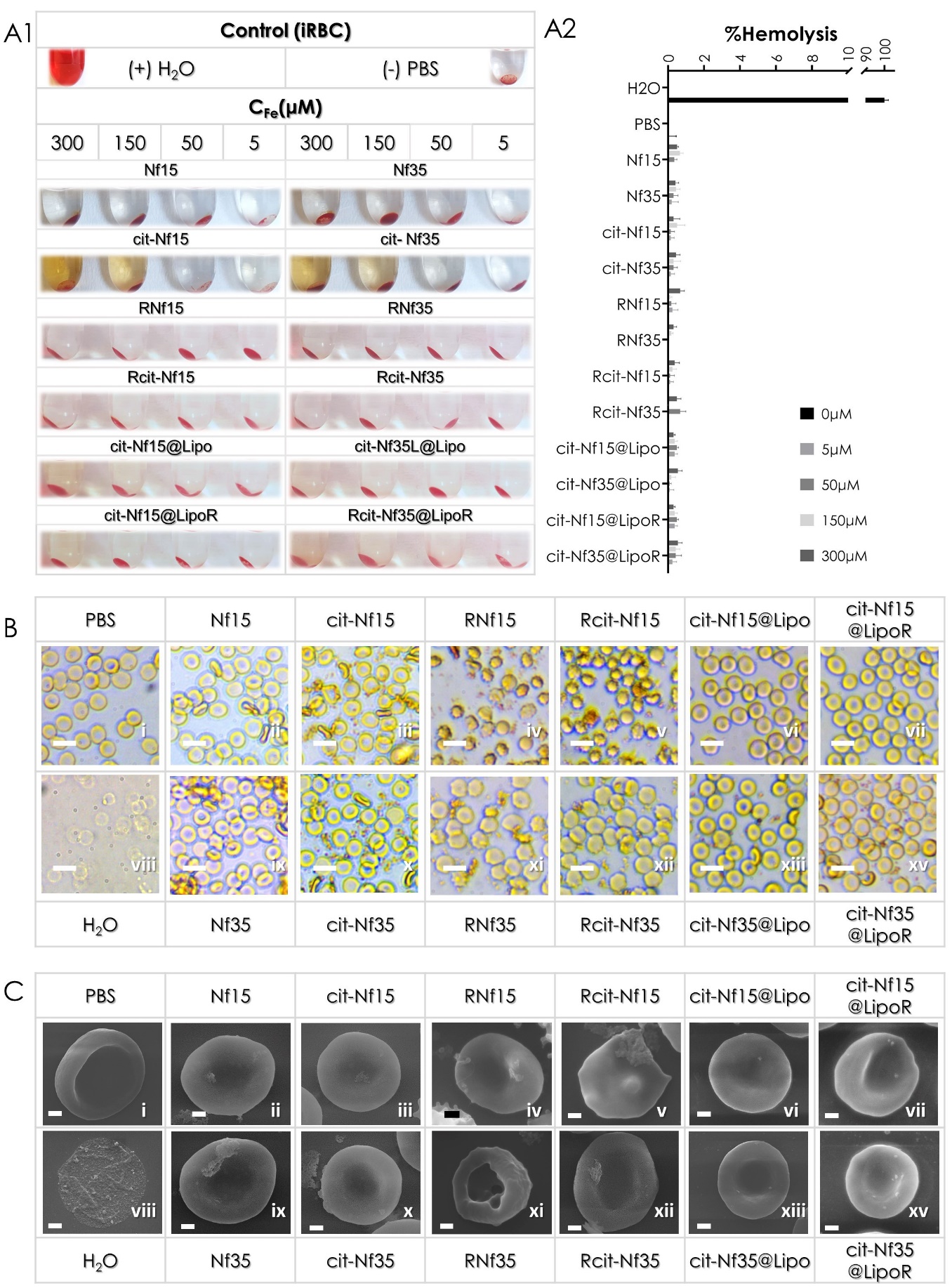


Figure S 3: Isolated RBCs: A1) Optical assessment of hemolysis and A2) hemoglobin percentage present in each supernatant after 3h incubation with the samples at different concentrations as measured in ELIZA, B) Optical microscopy (scale bars at 10μm) and C) SEM images (scale bars at 1μm) of RBCs after 3h incubation with the samples at a selected concentration (150μΜ) on coverslips in 24-well plates.


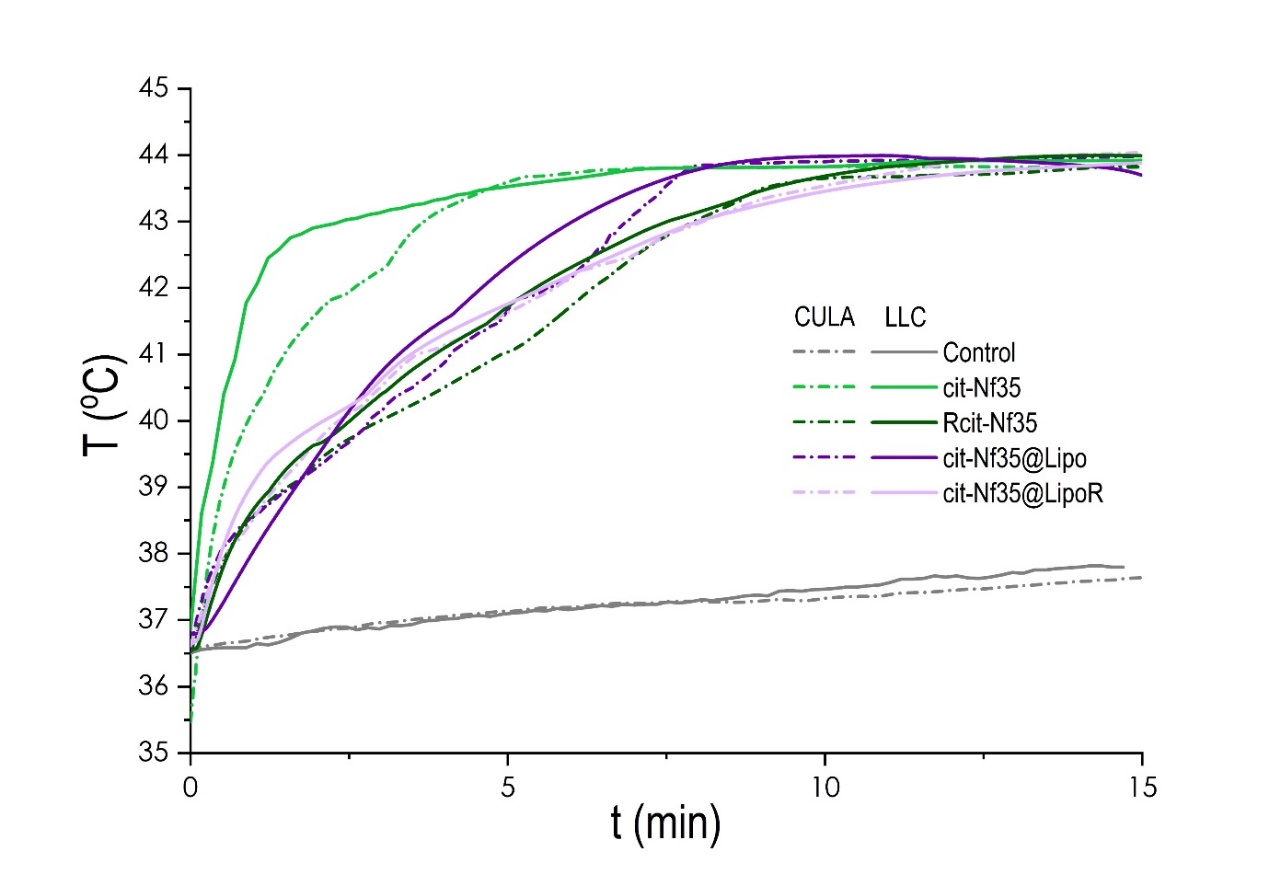


Figure S 4: Magnetic hyperthermia heating curves from in vitro experiments of all treated samples and control.


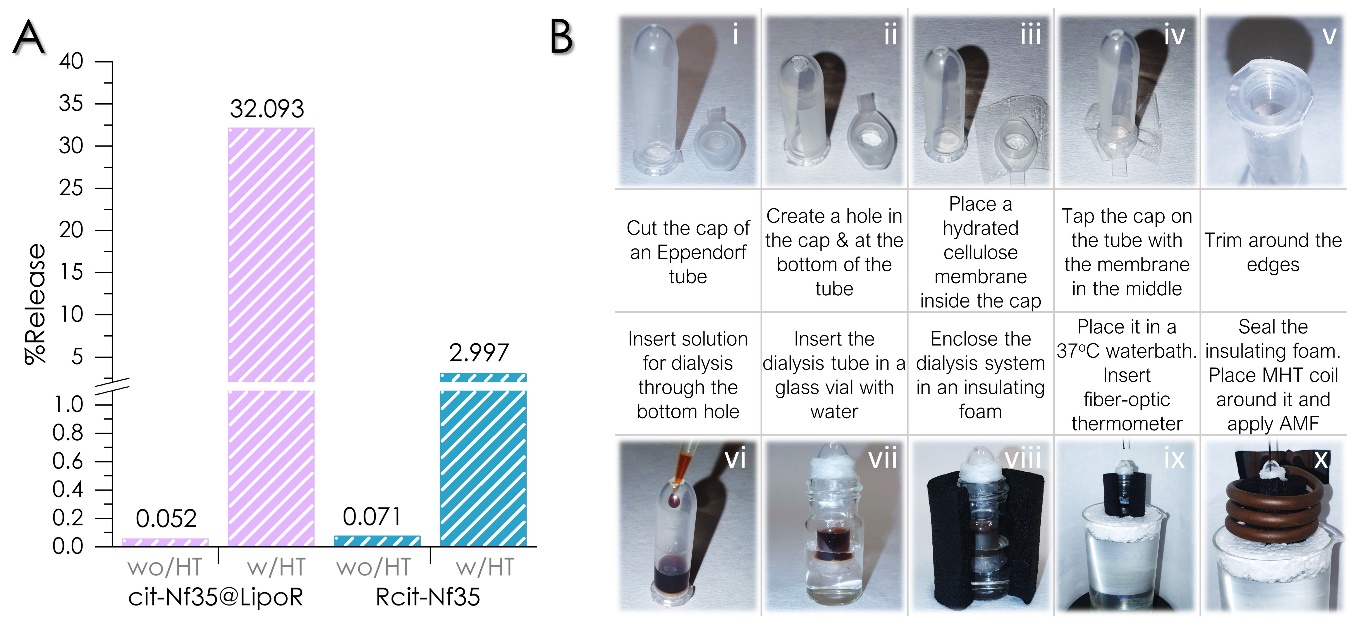


Figure S 5: A) RhB release percentage with or without hyperthermia in solution. B) Custom made set-up for the release experiment via dialysis under HT in adiabatic conditions.


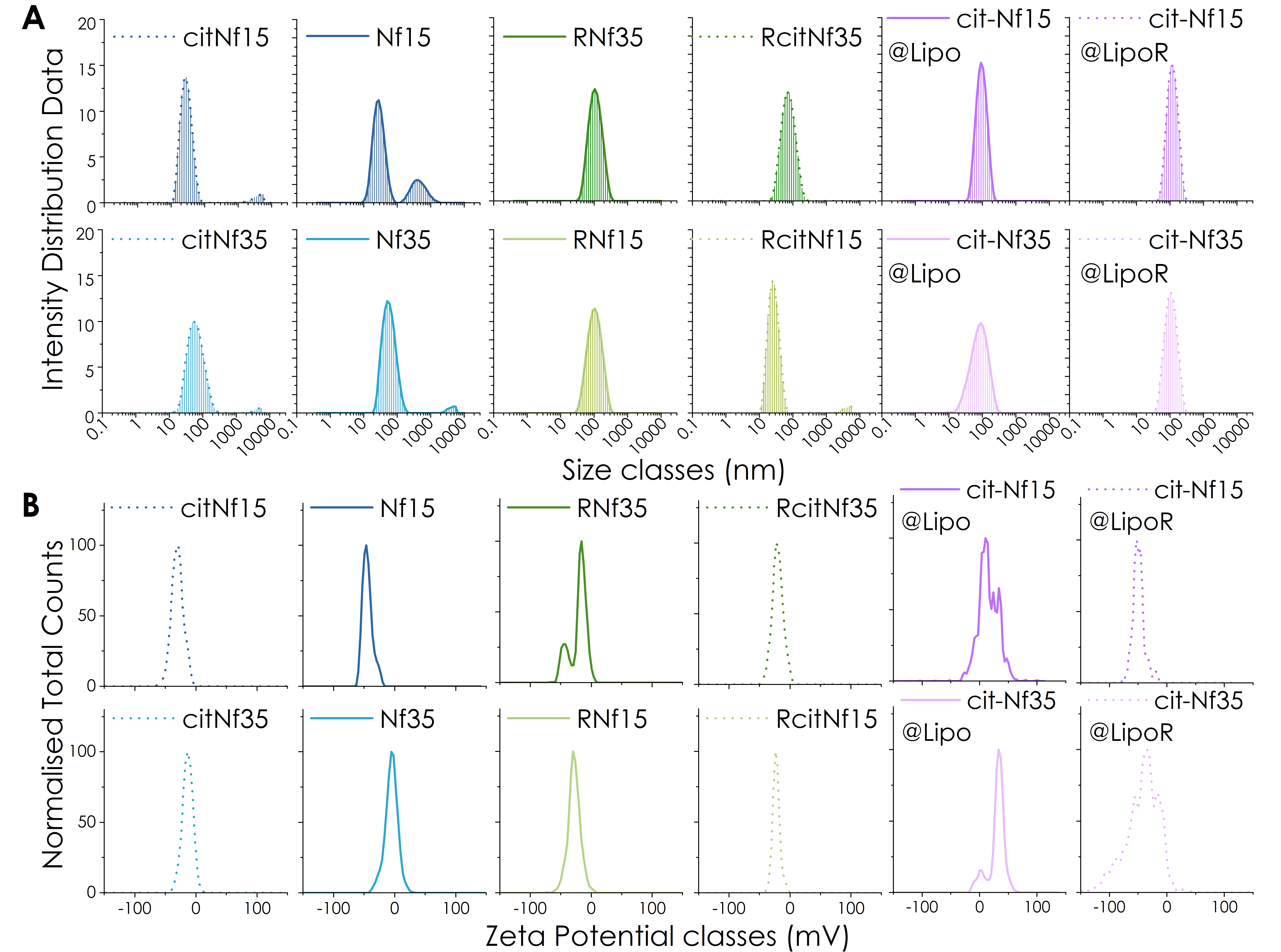


Figure S 6: Comparative DLS measurements of A) size distribution by intensity and B) ζ potential, for all synthesized formulations at 0.05 mg_Fe_/ml.


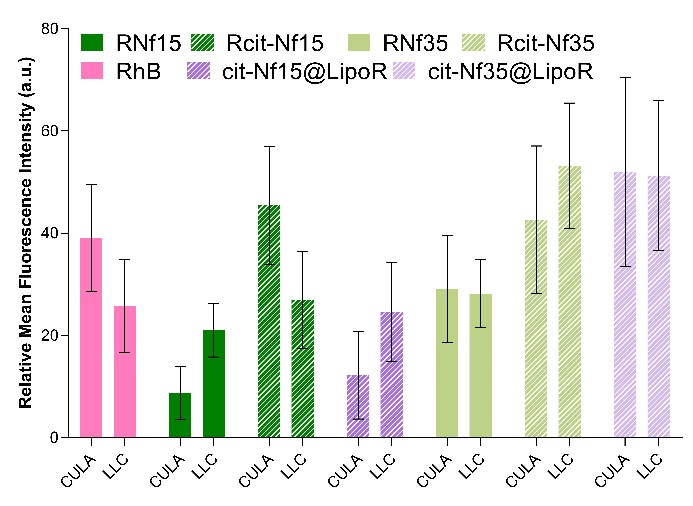


Figure S 7: Each bar represents the Relative Mean Fluorescence Intensity calculated from the images of fluorescent microscopy presented in Fig.6, with the image processing software ImageJ. The background is subtracted from the signal intensity and the ±SD value corresponds to the deviation of fluorescent intensity within each fluorescent region of interest.


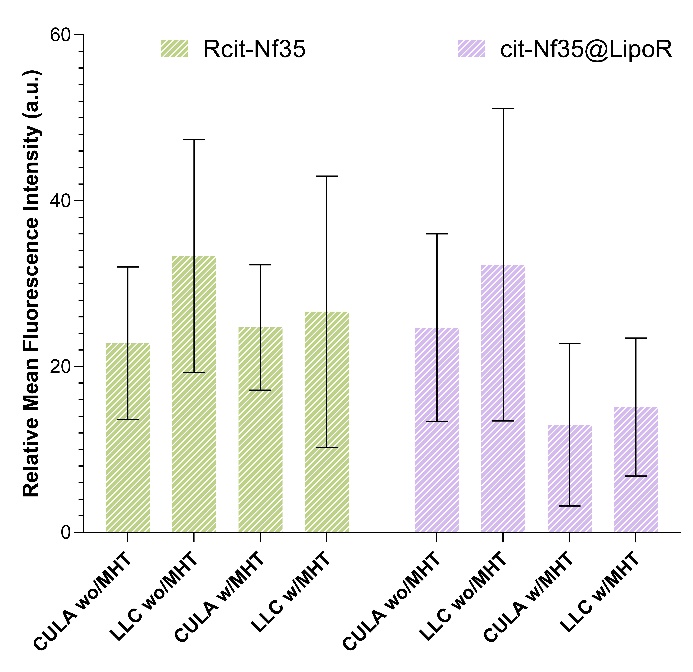


Figure S 8: Each bar represents the Relative Mean Fluorescence Intensity calculated from the images of fluorescent microscopy presented in Fig.7A, with the image processing software ImageJ. The background is subtracted from the signal intensity and the ±SD value corresponds to the deviation of fluorescent intensity within each fluorescent region of interest.

Table S 1: Remaining iron concentration in the LLC cell pellet after in vitro MHT experiment

|  | wo/MHT | | w/MHT | |
| --- | --- | --- | --- | --- |
|  | C_Fe_ (mM) | % C_Fe_ | C_Fe_ (mM) | % C_Fe_ |
| cit-Nf35 | 11.48 | 42.51 | 12.29 | 45.53 |
| cit-Nf35@Lipo | 11.30 | 41.85 | 12.75 | 47.24 |
| cit-Nf35@LipoR | 11.96 | 44.30 | 14.63 | 54.18 |
| Rcit-Nf35 | 13.09 | 48.48 | 16.68 | 61.76 |
